# Electrochemical Biosensing Interface Based on Carbon Dots-Fe_3_O_4_ Nanomaterial for the Determination of *Escherichia coli* O157:H7

**DOI:** 10.3389/fchem.2021.769648

**Published:** 2021-11-19

**Authors:** Xiaofeng Lin, Yanqiu Mei, Chen He, Yan Luo, Min Yang, Ying Kuang, Xiaoming Ma, Huifang Zhang, Qitong Huang

**Affiliations:** ^1^ Key Laboratory of Prevention and Treatment of Cardiovascular and Cerebrovascular Diseases, Ministry of Education, Key Laboratory of Biomaterials and Biofabrication in Tissue Engineering of Jiangxi Province, Oil-tea in Medical Health Care and Functional Product Development Engineering Research Center in Jiangxi, School of Public Health and Health Management, School of Pharmacy, School of Medical and Information Engineering, The Science Research Center, Gannan Medical University, Ganzhou, China; ^2^ School of Chemistry and Chemical Engineering, Key Laboratory of Organo-pharmaceutical Chemistry of Jiangxi Province, Gannan Normal University, Ganzhou, China

**Keywords:** *Escherichia coli* O157:H7, electrochemical biosensing interface, carbon dots, Fe_3_O_4_ nanomaterial, DNA biosensor

## Abstract

*Escherichia coli* (*E. coli*) O157:H7 can cause many food safety incidents, which seriously affect human health and economic development. Therefore, the sensitive, accurate, and rapid determination of *E. coli* O157:H7 is of great significance for preventing the outbreak and spread of foodborne diseases. In this study, a carbon dots-Fe_3_O_4_ nanomaterial (CDs-Fe_3_O_4_)-based sensitive electrochemical biosensor for *E. coli* O157:H7 detection was developed. The CDs have good electrical conductivity, and the surface of carbon dots contains abundant carboxyl groups, which can be used to immobilize probe DNA. Meanwhile, the CDs can be used as a reducing agent to prepare CDs-Fe_3_O_4_ nanomaterial. The Fe_3_O_4_ nanomaterial can improve the performance of the electrochemical biosensor; it also can realize the recovery of CDs-Fe_3_O_4_ due to its magnetism. As expected, the electrochemical biosensor has excellent specificity of *E. coli* O157:H7 among other bacteria. The electrochemical biosensor also exhibited good performance for detecting *E. coli* O157:H7 with the detection range of 10–10^8^ CFU/ml, and the detection limit of this electrochemical biosensor was 6.88 CFU/ml (3S/N). Furthermore, this electrochemical biosensor was successfully used for monitoring *E. coli* O157:H7 in milk and water samples, indicating that this electrochemical biosensor has good application prospect. More importantly, this research can provide a new idea for the detection of other bacteria and viruses.

## Introduction

In recent years, food poisoning caused by foodborne pathogens has frequently occurred worldwide, which is the most prominent public health problem in the world (Hou et al., 2020; Duan et al., 2021). As one of the most hazardous foodborne pathogens, *Escherichia coli* (*E. coli*) O157:H7 can cause some diseases such as hemorrhagic colitis and hemolytic uremic syndrome. What is more, low-dose *E. coli* O157:H7 can make humans sick, and in severe cases, it can even cause kidney failure and death. Currently, more than two million acute foodborne diseases worldwide are attributed to *E. coli* O157:H7 each year (Li et al., 2015a; Zhou et al., 2020). Therefore, it is very important to monitor the *E. coli* O157:H7 with high sensitivity. At present, some bioanalytical methods have been applied for *E. coli* O157:H7 detection, including enzyme-linked immunosorbent assay (ELISA) (Guo et al., 2016; Zhao et al., 2020), lateral flow immunochromatographic detection technology (Xie et al., 2014; Ye et al., 2020), polymerase chain reaction (PCR) (Dong et al., 2015), and so on. These alternative assays are the most rapidly used tests because of their good specificity and stability. Nevertheless, they are still faced with some limitations in detecting foodborne pathogens, including low sensitivity, complicated pretreatment, and high environmental impact. Therefore, there is an urgent need to establish a sensitive, simple, and highly specific method for detecting *E. coli* O157:H7. At present, many different electrochemical sensors have been used in food safety due to their some advantages, such as high sensitivity, economy, and accuracy (Li et al., 2015b; Li et al., 2016; Liu Y. et al., 2020; Dong et al., 2020). Therefore, electrochemical biosensors for the detection of *E. coli* O157:H7 have received extensive attention.

As is known, due to the excellent chemical, physical and biological properties of nanomaterials, they have been widely used in biomedical (Liu H. et al., 2020; Kuang et al., 2020; Thakur et al., 2020; Wu et al., 2020; Yi et al., 2021), analytical science (Chen et al., 2018; Broza et al., 2019; Jia et al., 2020; Lee et al., 2020; Mao et al., 2020; He S. et al., 2021; Mao et al., 2021), energy catalytic (Zhuang et al., 2019a; Zhuang et al., 2019b; Enesca, 2020; Zhuang et al., 2021), and other fields. Till now, some nanomaterials have been successfully used to construct electrochemical sensors for *E. coli* O157:H7 detection, such as graphene/AuNPs (You et al., 2020), reduced graphene oxide/polyaniline/Au@Pt/neutral red (rGO/PANI/Au@Pt/Nr) (Mo et al., 2019), rGO/Au@Pt (Zhu et al., 2018), rGO-poly (vinyl alcohol)/gold nanoparticles nanocomposite (AuNPs/rGO-PVA) (Qaanei et al., 2021), and so on. Although the abovementioned materials have made certain contributions to electrochemical sensors for detecting *E. coli* O157:H7, the abovementioned materials still have disadvantages, including complicated preparation, insufficient economy, and environmental protection, or low sensitivity of sensors. Therefore, it is important to prepare an easy-to-obtain, economical, and environmentally friendly functional material for the construction of electrochemical sensors for detecting *E. coli* O157:H7. As a member of “zero-dimensional” carbon nanomaterials, carbon dots (CDs) have some advantages of good biocompatibility, simple synthesis, and excellent conductivity (Zhang et al., 2019; Huang et al., 2020; Zhang and Du, 2020; He H. et al., 2021; Lin et al., 2021; Xu and Liu, 2021), and they have been successfully used to construct new electrochemical sensors, such as Pd-Au@CDs nanocomposite that we prepared for the preparation of the novel electrochemical DNA biosensor (Huang et al., 2017). Liu et al. designed an electrochemical sensor for measuring catechol based on F, N-doped CDs (Liu et al., 2019). Giang et al. constructed a wireless electrochemical biosensor based on CDs-hyaluronic acid/TiO_2_/Cu^2+^ for cancer cell detection (Giang et al., 2021). Sri et al. developed a CD/polymethyl methacrylate-based electrochemical biosensor for monitoring TNF-α targeting (Sri et al., 2021).

**FIGURE 1:**
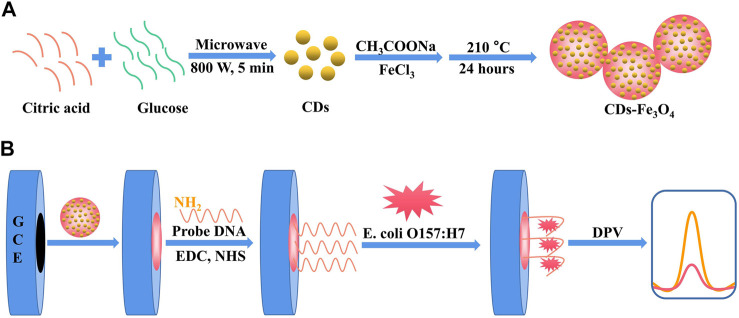
A sensitive electrochemical DNA biosensor based on CDs/Fe_3_O_4_ nanocomposite is constructed for *E. coli* O157:H7 detection.

In this study, a CDs-Fe_3_O_4_ nanomaterial-based electrochemical biosensor is constructed for detecting *E. coli* O157:H7, which is highly sensitive and reliable (Figure 1). The CDs have good electrical conductivity and large specific surface areas to improve the sensitivity of the sensor. The CDs also can be used as the reducing agent to synthesize the CDs-Fe_3_O_4_ nanomaterial. The Fe_3_O_4_ can improve the catalytic performance of the electrochemical biosensor, and it also can realize the recovery of CDs-Fe_3_O_4_ due to its magnetism. This electrochemical biosensor can achieve a good linear and detection limit for monitoring *E. coli* O157:H7. In addition, the biosensor has been also successfully applied to measure the *E. coli* O157:H7 in milk, indicating that the electrochemical biosensor has a broad application prospect. What is more, this study can provide a new idea for the preparation of electrochemical biosensors for other bacteria and virus detection, such as *Staphylococcus aureus* (*S. aureus*), *Listeria*, SARS-CoV-2, Hepatitis B virus, etc.

## Experimental

### Synthesis of carbon dots Fe_3_O_4_ nanomaterial

The preparation of CDs-Fe_3_O_4_ nanocomposite was based on the method of instrument preparation of our group (Kuang et al., 2020): First, 3 g of citric acid and 1 g of glucose were added in 5 ml H_2_O and reacted under microwave with a radiation power of 900 W for 6 min to synthesize CDs. Second, the above solution was cooled and added 30 ml H_2_O, centrifuged them at 12,000 rpm for 15 min and took the supernatant, then dialyzed the supernatant for 24 h and then freeze dried. Third, the abovementioned CD solid was dissolved in 25 ml of the aqueous solution, then 1 g of sodium acetate and 1 g of ferric chloride was added into 15 ml of CD solution, the mixed solution was sonicated for 8 h, then placed in an autoclave, and reacted at 210°C for 24 h; after cooling, they were centrifuged at 12,000 rpm for 10 min to acquire CDs-Fe_3_O_4_ nanomaterial.

### Preparation of electrodes

First, 5.0 μl of CDs-Fe_3_O_4_ nanomaterial was slowly dropped on the glassy surface of the carbon electrode (GCE) to prepare a CDs-Fe_3_O_4_/GCE working electrode. Second, the CDs-Fe_3_O_4_/GCE electrode was immersed in 1 ml of 0.1 mM PBS, 8.00 mM EDC, and 8.00 mM NHS mixed solution for 3 h to activate the electrode, then it was rinsed with TE buffer solution. Third, the above electrode was immersed in 1 ml of 0.1 μM probe DNA to react for 24 h, and it was rinsed with TE buffer solution to prepare the DNA/CDs-Fe_3_O_4_/GCE probe electrode. Finally, the prepared electrode was stored at 4°C.

### Preparation of the biosensor

The prepared DNA/CDs-Fe_3_O_4_/GCE was immersed into different concentrations (*C*) of *E. coli* O157:H7 solutions and reacted for 40 min at 42°C in a water bath. After the reaction, TE buffer solution was used to wash off non-specifically adsorbed *E. coli* on the surface to obtain the *E. coli* O157:H7-DNA/CDs-Fe_3_O_4_/GCE. Cyclic voltammetry (CV) and differential pulse voltammetry (DPV) have characterized the layer-by-layer assembly of each modified electrode: the [Fe (CN)_6_]^3-/4-^ has been applied as the electrolyte. For the test parameters of CV, the potential scanning range was −0.2–0.8 V, and the scan rate(*5*) was 0.1 V/s. DPV was used to record the electrochemical signal changes before and after the identification of the electrode and different substances, and the potential interval was −0.2–0.8 V, the amplitude was 0.05 V, the pulse width was 0.05 s.

## Results and discussion

### Characterization of nanomaterial

**FIGURE 2:**
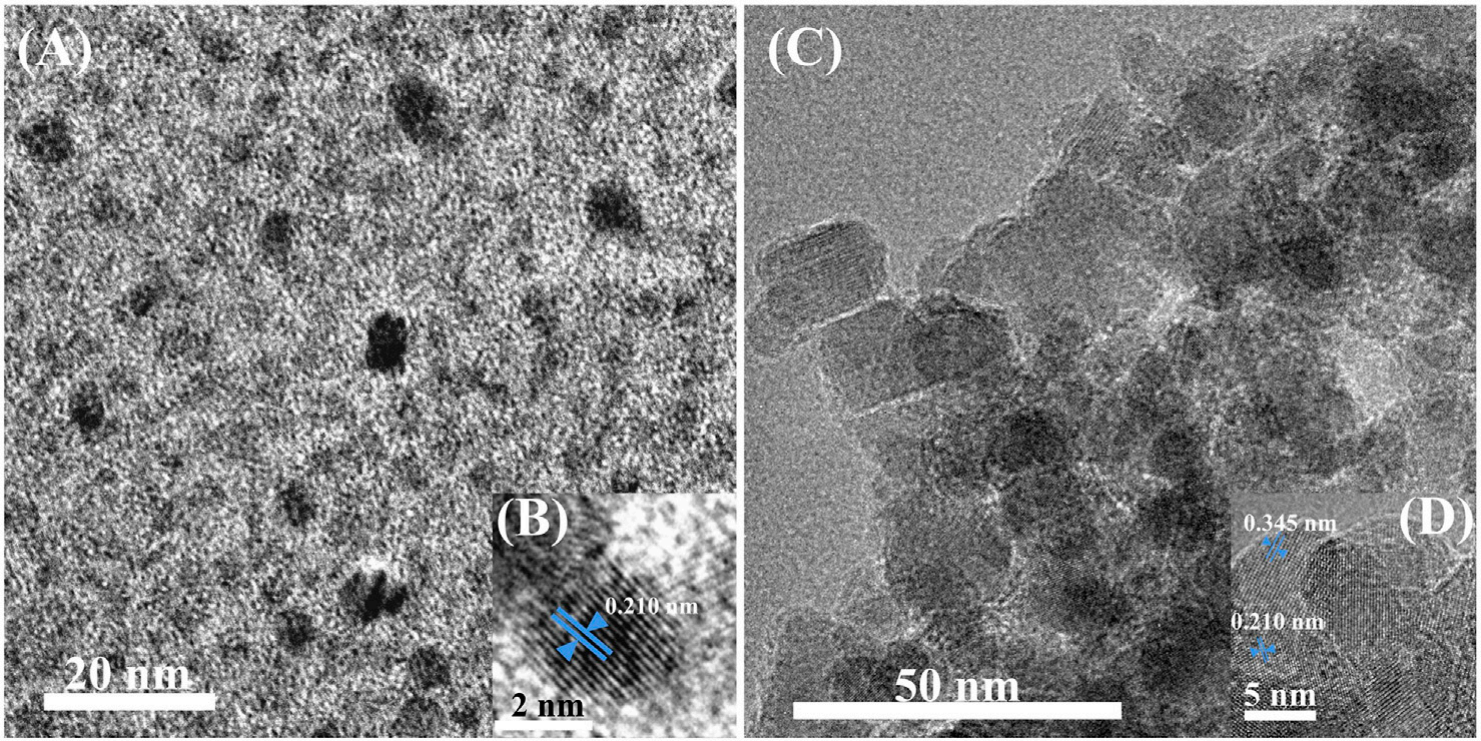
TEM and HRTEM image of CDs **(A,B)** and carbon dots (CDs)-Fe_3_O_4_ nanomaterial **(C,D)**.

Transmission electron microscopy (TEM) has characterized the morphologies of CDs and CDs-Fe_3_O_4_ nanomaterial. As shown in Figure 2A, the particle size distribution of the prepared CDs is relatively uniform, mainly at 3–4 nm. Figure 2B shows the high-resolution TEM (HRTEM) image of CDs. It shows that CDs have a good crystal lattice, and the lattice spacing is 0.210 nm, which is a typical (002) carbon crystal plane. As shown in Figure 2C, the particle size of the CDs-Fe_3_O_4_ nanomaterial was in the range of 15–40 nm. The HRTEM characterization of CDs-Fe_3_O_4_ (Figure 2D) shows that the nanomaterial has two lattice spacings, 0.345 and 0.210 nm, respectively. The corresponding crystal planes are (220) of the Fe_3_O_4_ and (002) of the carbon. The above morphology characterization can indicate that CDs-Fe_3_O_4_ nanomaterial has been successfully prepared.

### Cyclic voltammetry behavior of electrodes

**FIGURE 3:**
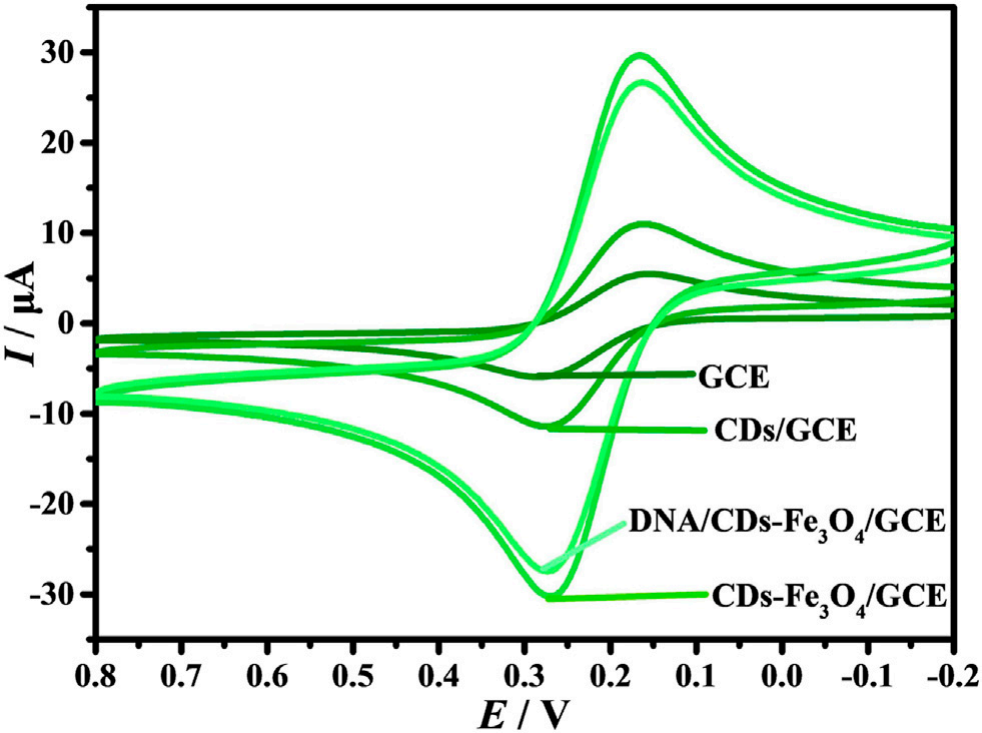
CVs of the electrodes in the 1.0 mM [Fe(CN)_6_]^3−/4−^ and 0.1 M KCl solution (*v*: 0.1 V/s).

It is well known that [Fe(CN)_6_]^3−/4−^ is often used as a supporting electrolyte for electrochemical biosensors (Fan et al., 2018; Li et al., 2021; Nano et al., 2021). The working electrodes were electrochemically characterized by CVs in 1.0 mM [Fe(CN)_6_]^3−/4−^ and 0.1 M KCl solution (Figure 3A). On the surface of GCE, the redox peak current (*I*) has good reversibility, and the oxidation peak potential (*I*_*pa*_) and reduction peak potential (*I*_*pc*_) are −6.616 and 5.958 A, respectively. When the CDs are modified on the surface of GCE, the *I*_*pa*_ and *I*_*pc*_ are promoted to −12.29 and 12.06 A, respectively, indicating that the prepared CDs have good conductivity. When the electrode is CDs-Fe_3_O_4_/GCE, the *I*_*pa*_ and *I*_*pc*_ are further enhanced, indicating that the introduction of Fe_3_O_4_ can improve the conductivity of the electrode. However, when the probe DNA is self-assembled on the surface of CDs-Fe_3_O_4_/GCE through carboxyammonia coupling reaction, the *I* decreases; the main reason is that the DNA molecules will hinder the diffusion of [Fe(CN)_6_]^3−/4−^ on the surface of CDs-Fe_3_O_4_/GCE. At the same time, the results indicate that the probe DNA was successfully modified on CDs-Fe_3_O_4_/GCE’s surface.

### Effects of scan rate

**FIGURE 4:**
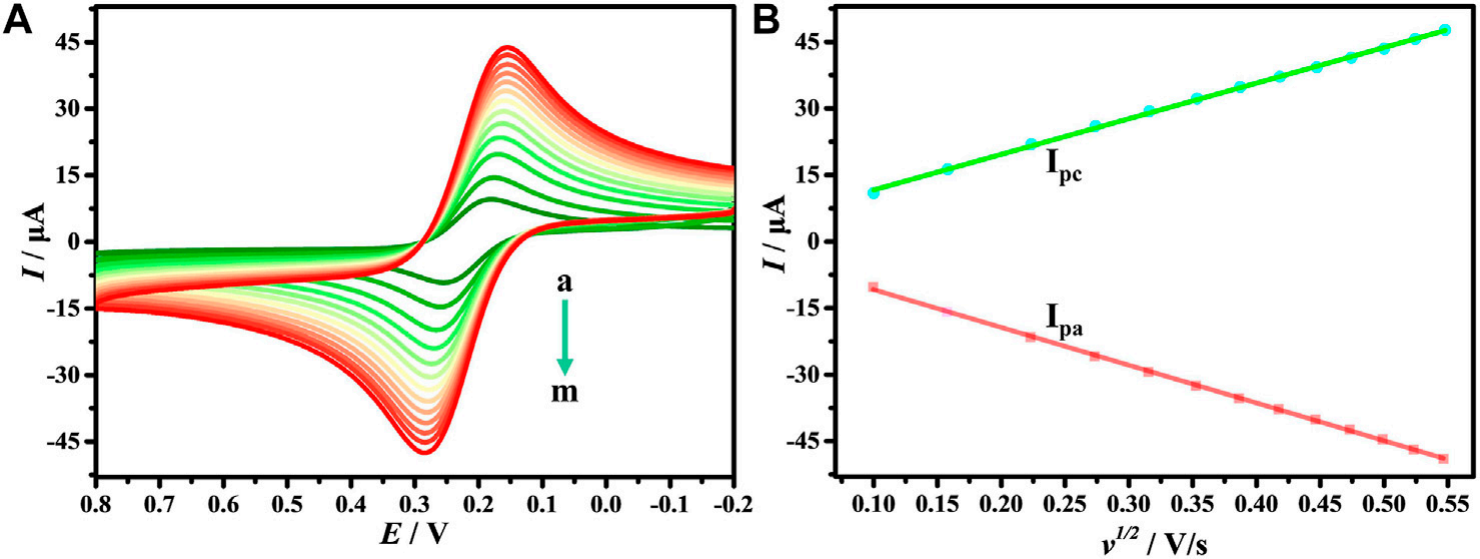
**(A)** CVs of DNA/CDs-Fe_3_O_4_/GCE in1.0 mM [Fe(CN)_6_]^3-/4-^ and 0.1 M KCl solution with different *v* (a–m: 0.01, 0.025, 0.05, 0.075, 0.1, 0.125, 0.15, 0.175, 0.20, 0.225, 0.25, 0,275, 0.3 Vs^−1^). **(B)** The relationship of redox peak current (*I*) with *v* ^*1/2*^.

The effect of different *v* on the electrochemical response of DNA/CDs-Fe_3_O_4_/GCE in [Fe(CN)_6_]^3−/4−^ has also been studied. Figure 4A shows that in the process of increasing *v*, the *I* also keeps increasing. As shown in Figure 4B, the *I*_*pa*_ and *I*_*pc*_ show an excellent linear relationship with *ν* ^*1/2*^, and the linear regression equations are *I*_*pa*_ = 3.598 + 80.265 *v* ^*1/2*^ and *I*_*pa*_ = −2.551–85.187 *v* ^*1/2*^, respectively. The results show that the electrochemical behavior of [Fe(CN)_6_]^3−/4−^ on the surface of DNA/CDs-Fe_3_O_4_/GCE is mainly affected by the diffusion-controlled process (Alam and Deen, 2020).

### Effect of interaction time

The interaction time between the probe electrode and the target substance is one of the key factors affecting the performance of the biosensors. Therefore, the interaction time of *E. coli* O157:H7 with DNA/CDs-Fe_3_O_4_/GCE was studied. When the *C* of *E. coli* O157:H7 was 10 CFU/Ml, the optimum hybridization time was determined to be 40 min (Supplementary Figure S1). However, when the *C* of *E. coli* O157:H7 was 10^8^ CFU/Ml, the optimum hybridization time was determined to be 35 min (Supplementary Figure S2). Therefore, in this electrochemical biosensor, the best reaction time between *E. coli* O157:H7 and DNA/CDs-Fe_3_O_4_/GCE is 40 min.

### Specificity of the biosensor

**FIGURE 5:**
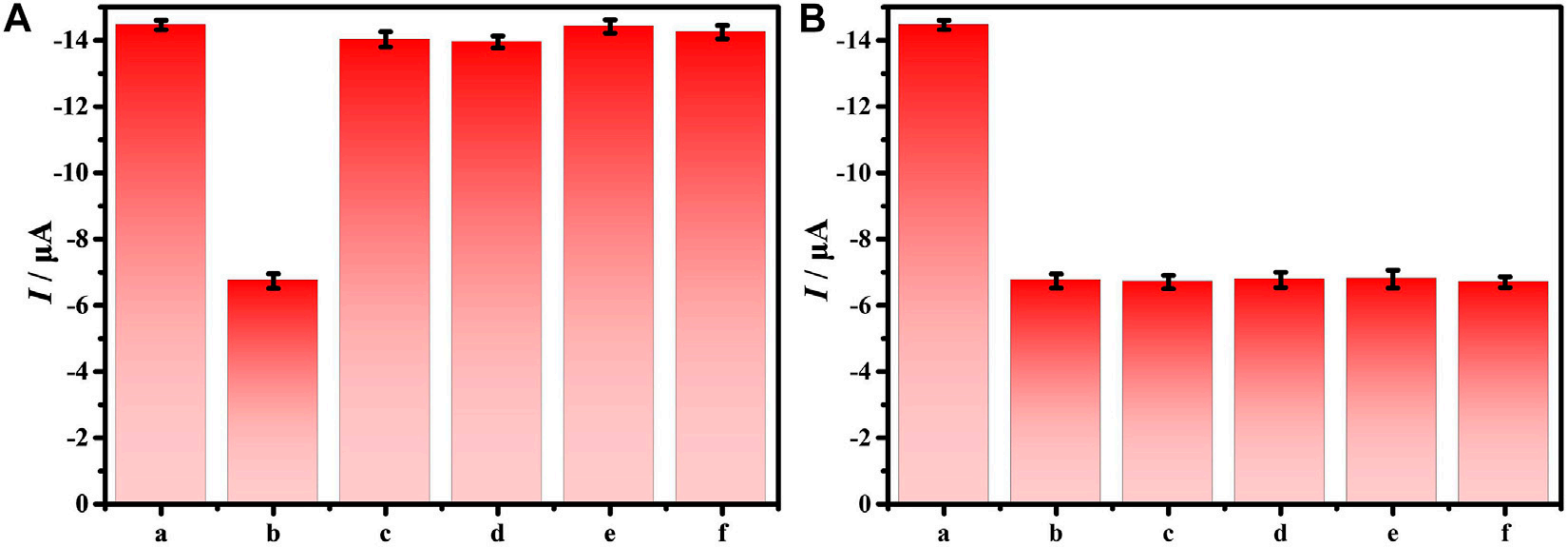
**(A)** *I_pa_* of 10^5^ CFU/ml different bacteria on the DNA/CDs-Fe_3_O_4_/GCE, **(B)** *I*_*pa*_ of 10^5^ CFU/ml *E. coli* O157:H7 with different bacteria on the DNA/CDs-Fe_3_O_4_/GCE (a: none, b: *E. coli* O157:H7, c: *S. aureus*, d: *Salmonella*, e: *S. lactis*, and f: *Listeria*).

The specificity of the electrochemical biosensor is a necessary factor to ensure the accuracy of the biosensor. Therefore, the interferences of different bacteria with 10^5^ CFU/ml on the DNA/CDs-Fe_3_O_4_/GCE through DPV have been investigated, such as *S. aureus*, *Salmonella*, *Staphylococcus lactis* (*S. lactis*), and *Listeria*. Figure 5A shows that DNA/CDs-Fe_3_O_4_/GCE has almost no effect on other bacteria. However, the DNA/CDs-Fe_3_O_4_/GCE has a very strong response to *E. coli* O157:H7, and the *I*_*pa*_ drops by 53.42%. As shown in Figure 5B, other interfering substances have almost no effect on the determination of *E. coli* O157:H7. The above results show that the electrochemical biosensor based on DNA/CDs-Fe_3_O_4_/GCE for detecting *E. coli* O157:H7 has very excellent specificity and is expected to realize the determination of *E. coli* O157:H7 in real samples.

### Sensitivity of the biosensor

**FIGURE 6:**
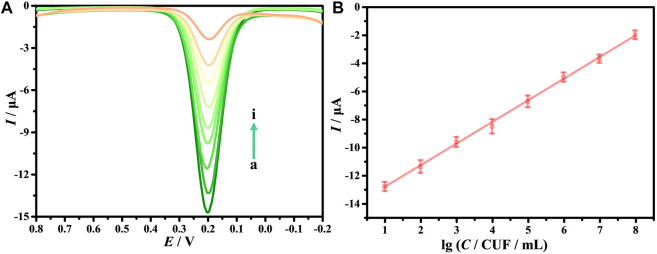
**(A)** Differential pulse voltammetry (DPV) of *E. coli* O157:H7 with different concentrations (from a to i: 0, 10, 10^2^, 10^3^, 10^4^, 10^5^, 10^6^, 10^7^, 10^8^ CFU/ml). **(B)** The relationship of *I*_*pa*_ with the lg*C*.

As is known, DPV has been extensively applied in the construction of electrochemical sensors because it has good detection sensitivity. This study explores the sensitivity and detection range of the electrochemical biosensors via DPV. Figure 6A shows that when *E. coli* O157:H7 interacts with DNA/CDs-Fe_3_O_4_/GCE, I_pa_ decreases as the *C* of *E. coli* O157:H7 increases. The main possible reason is *E. coli* O157:H7 has poor electrical conductivity, which affects the electrical conductivity of the working electrode’s interface, leading to a weakening of the electrochemical signal. When the *C of E. coli* O157:H7 is in the range of 10–107 CFU/ml, the lg*C* and *I*_*pa*_ show a good linear relationship (Figure 6B): *I*_*pa*_ = 1.5422 lg*C*—14.387. At the same time, the detection limit (LOD) can reach 6.88 CFU/ml. Compared with other electrochemical biosensors for monitoring *E. coli* O157:H7 (Table 1), this electrochemical biosensor based on DNA/CDs-Fe_3_O_4_/GCE has very good sensitivity.

**TABLE 1:** Electrochemical biosensors for detecting *Escherichia* (*E.) coli* O157:H7.

Materials	Methods	Detection ranges (CFU/ml)	LOD (CFU/ml)	References
Graphene/AuNPs/array electrode	EIS	10^2^–10^8^	10^2^	You et al. (2020)
rGO/Au@Pt/Screen printed carbon electrode	CV	4 × 10^3^–4 × 10^8^	4.5 × 10^2^	Zhu et al. (2018)
rGO/PANI/Au@Pt/Nr/Screen printed carbon electrode	CV	8.9 × 10^3^–8.9 × 10^9^	2.84 × 10^3^	Mo et al. (2019)
AuNPs/rGO-PVA/GCE	DPV	9.2–9.2 × 10^8^	9.34	Qaanei et al. (2021)
AuNPs/3D DNA walker/GCE	DPV	10–10^4^	7	Li et al. (2020)
Cu_3_(PO_4_)_2_/methylene blue/Gold electrode	DPV	10^2^–10^7^	32	Bu et al. (2020)
Cocoon-like DNA nanostructures/Gold electrode	DPV	10–10^6^	10	Bai et al. (2020)
DNA/CDs-Fe_3_O_4_/GCE	DPV	10–10^8^	6.88	This work

### Repeatability and stability of the biosensor

To investigate the reproducibility of this biosensor, six DNA/CDs-Fe_3_O_4_/GCE working electrodes were prepared, and they have been applied to detect 10^5^ CFU/ml of *E. coli* O157:H7 respectively. Supplementary Figure S3 shows that the relative standard deviation (RSD) was 2.6%, which indicates the electrochemical biosensor based on DNA/CDs-Fe_3_O_4_/GCE for *E. coli* O157:H7 detection has good reproducibility. The stability of the electrochemical biosensor has been also studied; the DNA/CDs-Fe_3_O_4_/GCE was placed in a dryer at 4°C, and the above working electrode was applied to measure 10^5^ CFU/ml of *E. coli* O157:H7 every 3 days. As shown in Supplementary Figure S4 after 30 days, the *I*_*pa*_ was only dropped by 2.7%, which shows that the electrochemical biosensor based on DNA/CDs- Fe_3_O_4_/GCE has good stability.

### Analysis of real samples

Since milk and water are often contaminated by *E. coli* O157:H7, it is very necessary to efficiently determine *E. coli* O157:H7 in milk and water. In this study, to verify the practical applicability of the biosensor, the electrochemical biosensor is used to determine the *E. coli* O157:H7 in milk and water. Table 2 shows that 10^3^, 10^4^, and 10^5^ CFU/ml of *E. coli* O157:H7 were added to the samples, and the recovery rates are between 95.2% and 102.0%, At the same time, the results were consistent with ELISA results, indicating that the method is feasible for monitoring *E. coli* O157:H7 in milk and water samples.

**TABLE 2:** Electrochemical biosensor for monitoring *E. coli* O157:H7 in milk and water samples.

Sample	Spike (CFU/ml)	Found (CFU/ml)	Recovery (%)	Detection by ELISA
Milk	10^3^	9.52 × 10^2^	95.2	9.46 × 10^2^
	10^4^	9.96 × 10^3^	99.6	9.53 × 10^3^
	10^5^	1.02 × 10^5^	102.0	9.91 × 10^4^
Water	10^3^	9.93 × 10^2^	99.3	9.86 × 10^2^
	10^4^	9.89 × 10^3^	98.9	9.81 × 10^3^
	10^5^	9.79 × 10^4^	97.9	9.69 × 10^4^

## Conclusions

In conclusion, a sensitive electrochemical DNA biosensor has been prepared for monitoring *E. coli* O157:H7 by CDs-Fe_3_O_4_ nanomaterial. Under the best conditions, when the *C* of *E. coli* O157:H7 is in the range of 10–10^8^ CFU/ml, the lg*C* has a good linear relationship with the *I*_*pa*_, and the LOD reaches 6.88 CFU/ml. At the same time, the electrochemical DNA biosensor has been successfully used to determine *E. coli* O157:H7 in milk and water samples. Therefore, this research provides an effective biosensor manufacturing strategy, which is expected to provide a reference for the preparation of electrochemical DNA biosensors for other bacteria or viruses detection.

## Data Availability

The original contributions presented in the study are included in the article/Supplementary Material. Further inquiries can be directed to the corresponding authors.
