# Supplementary material for: Electrochemical Biosensing Interface Based on Carbon Dots-Fe3O4 Nanomaterial for the Determination of Escherichia coli O157:H7
Source: Front Chem. 2021 Nov 19;9:769648. doi: 10.3389/fchem.2021.769648 (PMC8640100; doi:10.3389/fchem.2021.769648)
Supplement: Supplementary file 1 [file DataSheet1.PDF]

Electrochemical Biosensing Interface Based on Carbon Dots-Fe_3O_4 Nanomaterial for the Determination of *Escherichia coli* O157:H7

Xiaofeng Lin[1], Yanqiu Mei[1], Chen He[1], Yan Luo[1], Min Yang[1], Ying Kuang[1], Xiaoming Ma[2], Huifang Zhang[2,*], Qitong Huang[1,*]

[1] *Key Laboratory of Prevention and Treatment of Cardiovascular and Cerebrovascular Diseases, Ministry of Education, Key Laboratory of Biomaterials and Biofabrication in Tissue Engineering of Jiangxi Province, Oil-tea in Medical Health Care and Functional Product Development Engineering Research Center in Jiangxi, School of Public Health and Health Management, School of Pharmacy, School of Medical and Information Engineering, the Science Research Center, Gannan Medical University, Ganzhou, 341000, China.*
[2] *School of Chemistry and Chemical Engineering, Key Laboratory of Organo-pharmaceutical Chemistry of Jiangxi Province, Gannan Normal University, Ganzhou, 341000, China*

Corresponding authors：**E-mail address**: zhanghuifang93@163.com (H. Zhang); hqt@gmu.edu.cn, hqtblue@163.com (Q. Huang).

Materials and reagents

$K_4[Fe(CN)_6]$, $K_3[Fe(CN)_6]$, $FeCl_3$, glucose, CH3COONa, citric acid, and sodium acetate were purchased from Xilong Chemical Co.; Ltd. Bovine serum albumin (BSA), 1-Ethyl-3-(3'-dimethylaminopropyl)carbodiimide (EDC) and N-Hydroxysuccinimide (NHS) were purchased from Sinopharm Chemical Reagent Co.; Ltd. *E. coli* O157:H7 were obtained from Guangdong Microbial Culture Collection Center. Probe DNA sequence (Zhou et al. 2020): 5'-NH_2-$(CH_2)_6$-CCG GAC GCT TAT GCC TTG CCA TCT ACA GAG CAG GTG TGA CGG-3' was obtained from Shenggong Bioengineering Co.; Ltd.

Electrochemical experiments were measured on an electrochemical workstation (CHI660E). Transmission electron microscopy (TEM) and high-resolution TEM (HRTEM) were carried out on JEM-100CX.

References

Zhou, S., Lu, C., Li, Y., Xue, L., Zhao, C., Tian, G., Bao, Y., Tang, L., Lin, J., and Zheng, J. (2020). Gold Nanobones Enhanced Ultrasensitive Surface-Enhanced Raman Scattering Aptasensor for Detecting Escherichia coli O157:H7. *ACS Sensors* 5**,** 588-596.

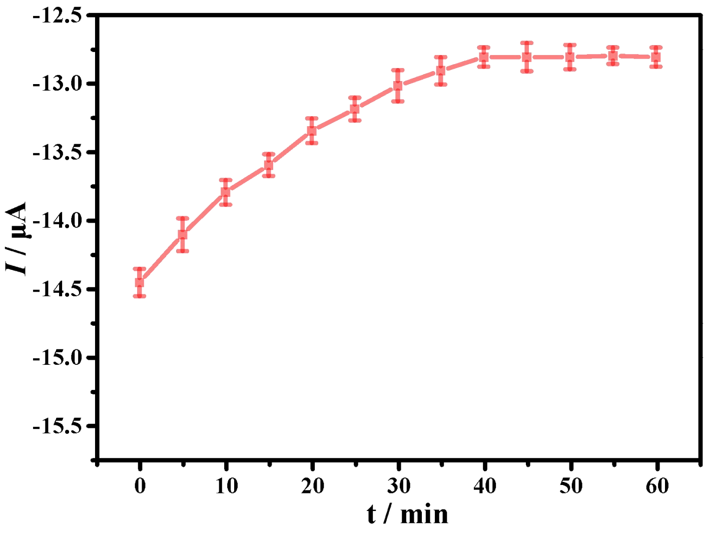

Figure S1 The reaction time of 10 CFU/mL *E. coli* O157:H7 with DNA/CDs-Fe_3O_4/GCE.

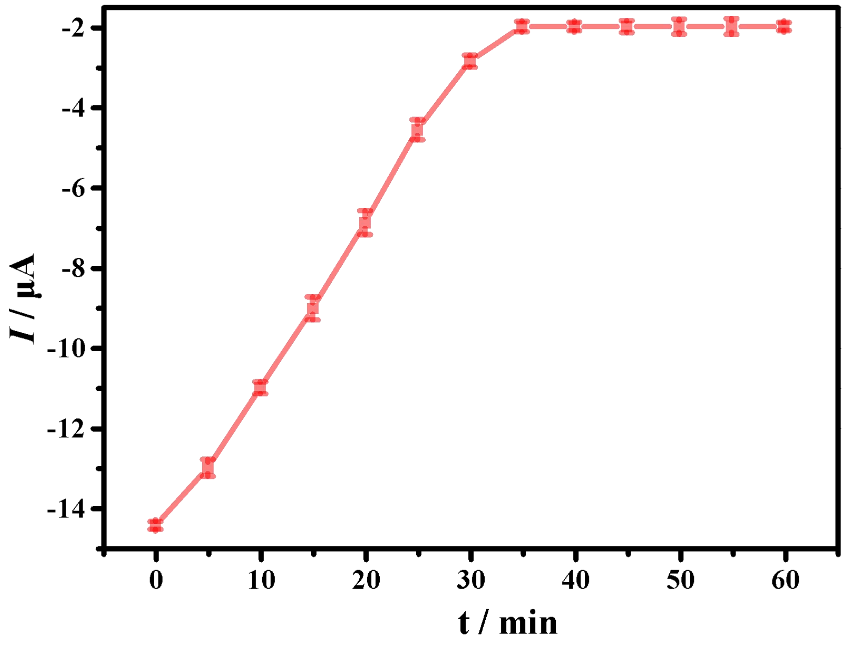

Figure S2 The reaction time of 10^8 CFU/mL *E. coli* O157:H7 with DNA/CDs-Fe_3O_4/GCE.

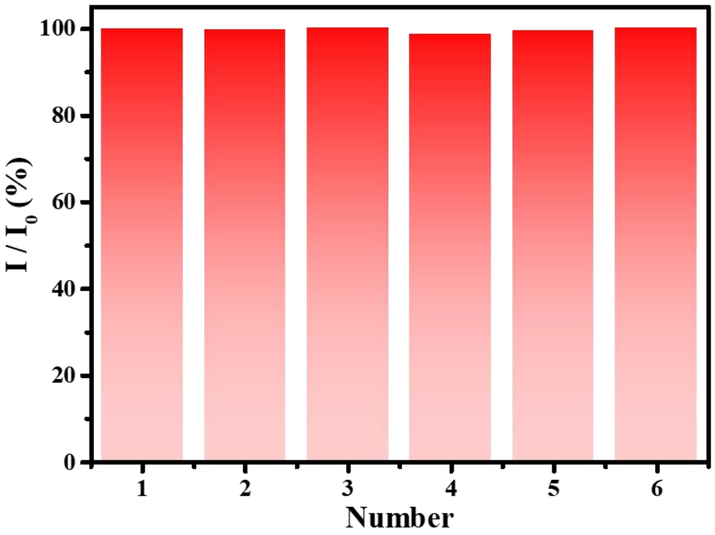

Figure S3 The repeatability of *E. coli* O157:H7 was determined by DNA/CDs-Fe_3O_4/GCE.

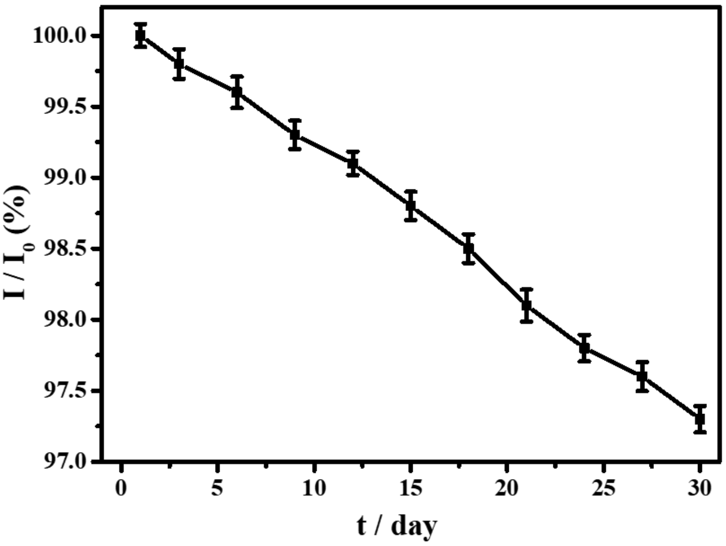

Figure S4 The stability of *E. coli* O157:H7 was determined by DNA/CDs-Fe_3O_4/GCE.
